# SAMA: A Fast Self-Adaptive Memetic Algorithm for Detecting SNP-SNP Interactions Associated with Disease

**DOI:** 10.1155/2020/5610658

**Published:** 2020-08-24

**Authors:** Ying Yin, Boxin Guan, Yuhai Zhao, Yuan Li

**Affiliations:** ^1^Key Laboratory of Intelligent Computing in Medical Image, Minister of Education, and School of Computer Science and Engineering, Northeastern University, Shenyang 110819, China; ^2^School of Information Science and Technology, North China University of Technology, Beijing 100144, China

## Abstract

Detecting SNP-SNP interactions associated with disease is significant in genome-wide association study (GWAS). Owing to intensive computational burden and diversity of disease models, existing methods have drawbacks on low detection power and long running time. To tackle these drawbacks, a fast self-adaptive memetic algorithm (SAMA) is proposed in this paper. In this method, the crossover, mutation, and selection of standard memetic algorithm are improved to make SAMA adapt to the detection of SNP-SNP interactions associated with disease. Furthermore, a self-adaptive local search algorithm is introduced to enhance the detecting power of the proposed method. SAMA is evaluated on a variety of simulated datasets and a real-world biological dataset, and a comparative study between it and the other four methods (FHSA-SED, AntEpiSeeker, IEACO, and DESeeker) that have been developed recently based on evolutionary algorithms is performed. The results of extensive experiments show that SAMA outperforms the other four compared methods in terms of detection power and running time.

## 1. Introduction

The development of high-throughput sequencing technology makes it possible to analyze single-nucleotide polymorphisms (SNPs) from thousands of individuals [1, 2]. With the purpose of detecting the association between SNPs and a disease, genome-wide association study (GWAS) plays a vital role in recognizing causes of diseases [3–5]. GWAS has been successfully applied to identify numerous SNPs associated with diverse diseases, such as about 30 loci associated with schizophrenia [6–8]. However, due to the large amount of computation imposed by the high-dimensional search space, it is difficult to measure the association between SNP-SNP interactions and disease in genome-wide data [9–11].

In the past few years, many methods have been raised for detecting two-locus disease models. These algorithms can be categorized into exhaustive search, stochastic search, heuristic search, and swarm intelligent optimization algorithms [12]. The exhaustive search is a method which evaluates the degree of correlation between all possible SNP-SNP interaction combinations and disease [13, 14] but is often computationally unaffordable for datasets with very large number of SNPs.

The random search uses probabilistic methods to find the optimal solution [15, 16]. The heuristic search is an approximate search algorithm that speeds up the search process by reducing the search space [17, 18]. However, the two kinds of searches cannot make the commitment of finding the optimal solution all the time.

In the recent years, swarm intelligent optimization algorithms arising from natural phenomena and biological system have held high attention in the detection of disease-associated SNP-SNP interactions [19–21]. For instance, FHSA-SED [22] combines the harmony search algorithm with two scoring functions for the detection of SNP-SNP interactions. AntEpiSeeker [23] detects disease-associated SNP-SNP interactions by using a two-stage ant colony optimization (ACO) [24, 25]. IEACO [26] automatically adjusts path selection strategies using information entropy to detect SNP-SNP interactions. DESeeker [27] uses a two-stage differential evolution (DE) [28, 29] algorithm to identify the SNP-SNP interaction. However, it is worth noticing that all of these methods remain defective owing to their low detection power.

One promising approach for tackling the drawbacks mentioned above is to use a fast local search in the evolutionary algorithm. Hybridization of genetic algorithms (GAs) with local search (LS) has already been studied in various optimization problems [30–32]. Such a hybrid algorithm is often called a memetic algorithm (MA) [33]. Thus, we propose a fast self-adaptive memetic algorithm (SAMA) to detect two-locus SNP-SNP interactions associated with disease. In the SAMA algorithm, we improve the crossover, mutation, and selection of MA. These three improved operations are more suitable for detecting two-locus SNP-SNP interactions. Moreover, we incorporate a self-adaptive local search into the proposed algorithm to avoid premature convergence. We compare our algorithm with the state-of-the-art methods and conduct experiments on a wide range of simulated datasets and a real-world biological dataset. The results show the proposed algorithm has improved power in detecting correct SNP-SNP interactions with different disease models.

The paper is organized as follows. In Section 2, we introduce the problem definition of two-locus SNP-SNP interactions associated with disease and propose the SAMA algorithm. In Section 3, we describe the experiments carried out in order to determine the detection power of our method. Finally, we present the conclusion in Section 4.

## 2. Methods

### 2.1. Problem Definition

A set of SNPs is represented by *S* = {*r*_1_, *r*_2_, ⋯, *r*_*L*_}, where *r* is an SNP and *L* is the number of SNPs. For detecting two-locus disease models, there are *L*(*L* − 1)/2 combinations that can be selected. The value of each SNP is 0, 1, or 2, which represent the homozygous major genotype, the heterozygous genotype, and the homozygous minor genotype, respectively. A dataset contains *n* samples (*n*_*d*_ cases and *n*_*u*_ controls), and each sample has a set of SNPs. If the genotype distribution of a two-locus SNP-SNP interaction is significantly different between cases and controls, it may lead to an increase in the risk of the disease.

### 2.2. The SAMA Algorithm

It is a time-consuming task to detect SNP-SNP interactions associated with disease if all possible two-locus interactions from hundreds of thousands of SNPs are considered in a genome-wide scale. In this paper, a fast self-adaptive memetic algorithm (SAMA) is proposed to enhance the detection power of two-locus SNP-SNP interactions in an efficient way.

**Figure 1:**
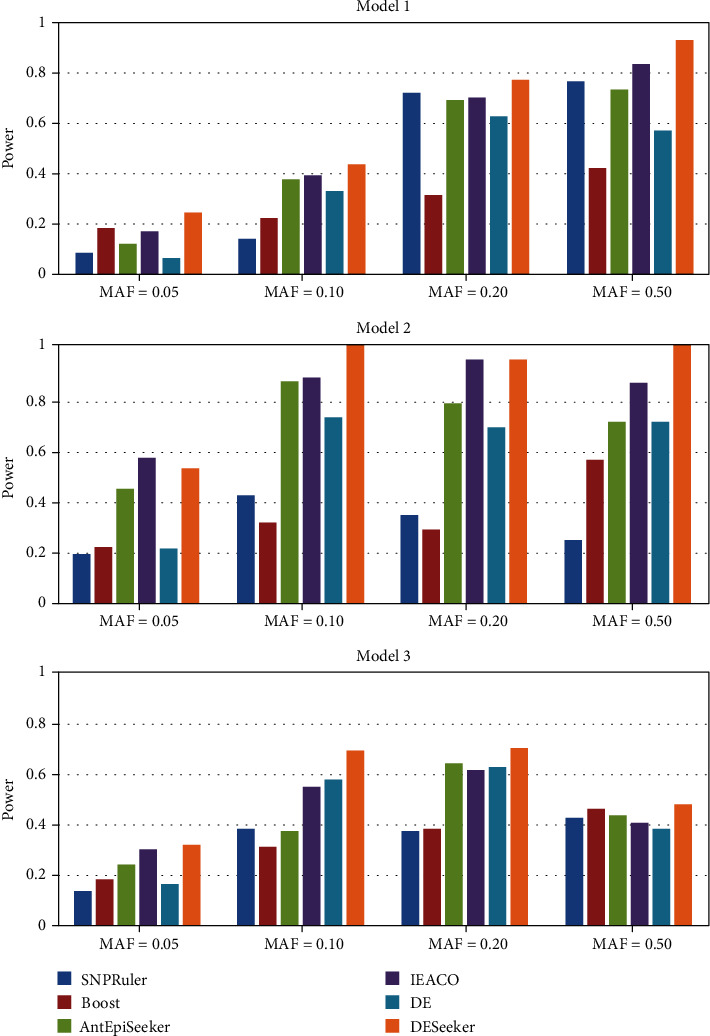
The framework of MA.

Memetic algorithm (MA) [33] is inspired by natural system model and population evolution. By combining evolutionary algorithms with local search, it can provide a local improvement opportunity for the individuals in a genetic search. The framework of MA can be outlined as Figure 1, and this figure shows the basic structure of the MA algorithm. MA consists of two parts: genetic search and local search, where the local search part includes crossover, mutation, and selection. The SAMA algorithm follows the basic framework in Figure 1 to detect two-locus SNP-SNP interactions associated with disease, and the process is shown in Algorithm 1.

#### 2.2.1. Initialization

The SAMA algorithm randomly generates a initial population with *M* individuals. An individual is expressed as *x*_*i*_ = {*r*_*p*_, *r*_*q*_}(1 < *r*_*p*_, *r*_*q*_ < *L*) where *r*_*p*_ and *r*_*q*_ are SNPs, and the individual *x*_*i*_ is generated by

(1) $$\begin{array}{c} x_{i}=\left\{r_{p}\leftarrow \left\lceil \mathrm{rand}\left(0,1\right)\cdot L\right\rceil ,r_{p}\leftarrow \left\lceil \mathrm{rand}\left(0,1\right)\cdot L\right\rceil \right\}, \end{array}$$

where ⌈ ⌉ is an upward rounding operation, rand(0, 1) is a random number between 0 and 1, and *L* is the number of SNPs in a dataset. After initialization, SAMA finds the current optimal solution *x*_best_ with the best value of fitness function. In SAMA, the *χ*^2^ test is used as the fitness function to measure the association between two-locus SNP-SNP interactions and the disease.

#### 2.2.2. Hybrid Crossover (HC)

The crossover operator, a fundamental genetic search operator, takes advantage of the information available in the search space. In the SAMA algorithm, we use a hybrid crossover (HS) to cross two individuals. HC can be considered the hybrid between the current best individual and the individuals in the current iteration. The pseudocode of HC is shown in Algorithm 2.

In the algorithm, the current best individual *x*_best_ and the individual *x*_*i*_ in the current iteration are selected as two parents. If the random number *r*1 between 0 and 1 is less than the crossover probability *p*_*c*1_, the first SNP *r*_*p*_ in *x*_*i*_ is replaced by the first SNP *r*_best*p*_ in *x*_best*p*_. If the random number *r*2 is less than the crossover probability *p*_*c*2_, the second SNP *r*_*q*_ in *x*_*i*_ is replaced by the second SNP *r*_best*q*_ in *x*_best*q*_. If the conditions of *r*_1_ < *p*_*c*1_ and *r*_2_ < *p*_*c*2_ are satisfied at the same time, *x*_*i*_ is replaced by *x*_best_.

#### 2.2.3. Distributed Breeder Mutation (DBM)

The mutation operator is used to randomly create the diversity of individuals in a population. We use a mutation called distributed breeder mutation (DBM) in the SAMA algorithm. DBM, inspired by the breeder genetic algorithm proposed by Muhlenbein and Schlierkamp-Voosen [34], is a robust global search based on a solid theory. The mutated individual *z*_*i*_ is calculated by the following equation:

(2) $$\begin{array}{c} \begin{array}{c} {\text{z}}_{\text{i}}=\left\{\text{r}\prime_{\text{p}}\leftarrow {\text{r}}_{\text{p}}\pm \text{range}\cdot \delta ,\;\text{r}\prime_{\text{q}}\leftarrow {\text{r}}_{\text{q}}\pm \text{range}\cdot \delta \right\}\\ {\text{r}}_{\text{p}}\;\text{and}\;{\text{r}}_{\text{p}}\in {\text{y}}_{\text{i}}, \end{array} \end{array}$$

where range is the mutation set to 0.1 · L, *δ* is calculated from a distribution which prefers a small value, and the “+” or “−” is chosen with a probability of 0.5. Thus, r_p_ is mutated in the interval between [r_p_ − range · *δ*] and [r_p_ + range · *δ*], and r_q_ is mutated in the interval between [r_q_ − range · *δ*] and [r_q_ + range · *δ*].

If the mutated individual z_i_ is outside the specified range (1 < r_p_, r_q_ < L), z_i_ will be reinitialized. *δ* is computed according to the following equation:

(3) $$\begin{array}{c} \delta =\overset{15}{\underset{\text{i}=1}{\sum }}\alpha_{\text{i}}2^{-\text{i}}\;\alpha_{\text{i}}\in \left(0,1\right). \end{array}$$

*α*_i_ is set to 0 before the mutation operation. Then, each *α*_i_ is mutated to 1 with a probability of 1/16. The minimum step size is produced with a precision of range_i_ · 2^−15^.  Algorithm 3 gives the execution process of DBM.

#### 2.2.4. Self-Adaptive Local Search (SLS)

Local search (LS) is a simple iterative method for finding approximate solutions. If a candidate solution has better or equal fitness, LS moves the search from the current solution to the candidate solution. If LS is applied to every solution many times, the running time is very long because the additional functional evaluations required for LS is expensive. Thus, a self-adaptive LS (SLS) is introduced, which uses a probability to reduce the number of times that are used for local search. The probability that each individual is selected to allpy the SLS operation is p_z_i__, and the p_z_i__ is defined by

(4) $$\begin{array}{c} {\text{p}}_{{\text{z}}_{\text{i}}}=\left(\begin{array}{cc} 1 & \text{if }{\text{z}}_{\text{i}}\text{ is improved}\\ \xi \cdot {\text{p}}_{{\text{z}}_{\text{i}}} & \text{otherwise}, \end{array}\right. \end{array}$$

where *ξ* is the switch parameter, and z_i_ is an individual after HC and DBM. The initial p_z_i__ of each individual is 1; hence, each individual will be selected at least once for SLS. If the fitness value of the individual z_i_ is improved, the probability p_z_i__ that z_i_ is selected is still 1. Otherwise, p_z_i__ is changed to *ξ* · p_z_i__. If the fitness value of z_i_ is not improved after being selected n times, this value is *ξ*^n^ · p_z_i__. The pseudocode of SLS is shown in Algorithm 4.

#### 2.2.5. Elitist Selection (ES)

In the SAMA algorithm, an elitist selection is introduced to select individuals that evolve to the next iteration. After HC, DBM, and SLS, the ES operation is performed according to

(5) $$\begin{array}{c} {\text{x}}_{\text{i}}=\left(\begin{array}{cc} {\text{w}}_{\text{i}} & \text{if fit}\left({\text{w}}_{\text{i}}\right)>\text{fit}\left({\text{x}}_{\text{i}}\right)\\ {\text{x}}_{\text{i}} & \text{if fit}\left({\text{w}}_{\text{i}}\right)\leq \text{fit}\left({\text{x}}_{\text{i}}\right). \end{array}\right. \end{array}$$

If the fitness value of the individual w_i_ is greater than that of the previous individual x_i_, x_i_ is replaced by w_i_. Otherwise, x_i_ is unchanged.

### 2.3. A Running Instance of SAMA

**Figure 2:**
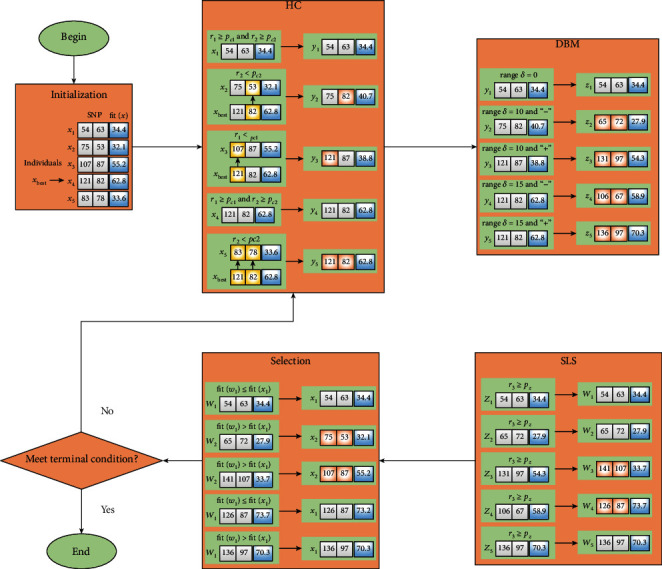
A running instance of SAMA.

In this subsection, we give a running instance of SAMA in Figure 2. Suppose that there are five individuals in the current population. After initialization, x_1_ = (54, 63), x_2_ = (75, 53), x_3_ = (107,87), x_4_ = (121,82), and x_5_ = (83, 78). Among them, x_4_ obtains the highest fitness value, i.e., fit(x_i_) = 62.8, and hence, x_4_ is the current optimal solution x_best_ (r_bestp_ = 121 and r_bestq_ = 82).

First, we perform the HC operation. Suppose r_1_≥p_c1_ and r_2_≥p_c2_ for x_1_ and x_4_, r_2_ < p_c2_ for x_2_, r_1_ < p_c1_ for x_3_, and r_1_ < p_c1_ and r_2_ < p_c2_ for x_5_. According to Algorithm 2, x_1_ and x_4_ are not changed and assigned directly to y_1_ and y_4_, whereas the other three individuals are changed. One SNP in x_2_ and x_3_ is replaced; hence, x_2_ is changed to y_2_ = (75, 82) and x_3_ is changed to y_3_ = (121,87). x_5_ is changed to y_5_ = (121,82) because both SNPs in x_5_ are replaced.

Next is the DBM operation. We assume that range · *δ* of y_1_ is 0, the range · *δ* of y_2_ and y_3_ is 10, and the range · *δ* of y_4_ and y_5_ is 15. y_2_ and y_4_ get “−”, whereas y_3_ and y_5_ get “+.” Thus, y_1_ is not changed and assigned directly to z_1_ = (54, 63), y_2_ is changed to z_2_ = (65, 72), y_3_ is changed to z_3_ = (131,97), y_4_ is changed to z_4_ = (106,67), and y_5_ is changed to z_5_ = (136,97).

After completing HC and DBM, the SLS operation is executed. z_1_, z_2_, and z_5_ are not changed and assigned directly to w_1_, w_2_, and w_5_ due to r3 ≥ p_z_. For z_3_ and z_4_, SLS is operated cyclically because of r3 < p_z_. z_3_ is changed to w_3_ = (141,107) and z_4_ is changed to w_4_ = (126,87) after the DMB operation in SLS.

Finally, the selection operation is performed. We suppose that fit(w_1_) ≤ fit(x_1_), fit(w_2_) ≤ fit(x_2_), fit(w_3_) ≤ fit(x_3_), fit(w_4_) > fit(x_4_), and fit(w_5_) > fit(x_5_). Thus, x_1_, x_2_, and x_3_ are retained to the next generation. For x_4_ and x_5_, the two individuals are replaced and assigned to the next generation.

## 3. Results

To evaluate of the performance of the SAMA algorithm, we test it on both simulated and real-world biological datasets. we compare it with FHSA-SED, AntEpiSeeker, IEACO, and DESeeker on these datasets. For the simulated datasets, we adopt three two-locus disease models. For the real-world biological dataset, we run SAMA on an age-related macular degeneration (AMD) data [35].

### 3.1. Simulated Datasets

In this subsection, we carry out the experiments in three simulated disease models (Models 1-3) [36]. Model 1 is a two-locus multiplicative model in which the disease prevalence (P(D)) increases multiplicatively with the incremental presence of the disease genotype interaction. Model 2 is a two-locus threshold model, in which P(D) does not increase until the number of disease genotype interactions pass the threshold. Model 3 is a two-locus concrete mode that simulates the effects of SNP-SNP interactions on susceptibility to traits. In the three models, P(D) is set to 0.1, and the minor allele frequencies (MAF) is 0.05, 0.10, 0.20, and 0.50. The genetic heritability (h^2^) is 0.005 in Model 1, and h^2^ is 0.02 in Models 2 and 3. According to the combination of these values, 12 penetrance tables are obtained (see Table 1). 200 datasets corresponding to each penetrance table are generated using GAMETES_2.0 [37]. 100 SNPs are generated in the first 100 datasets, whereas the number of SNPs is 2000 in the other 100 datasets.

### 3.2. Parameter Setting

In the experiments, we set the same maximum number of iterations for the five algorithms, that is, the maximum iteration number for datasets with 200 SNPs is set to 50 and the maximum iteration number for datasets with 2000 is set to 500. The maximum number of iterations is less than the number of iterations using an exhaustive algorithm. Furthermore, the other parameters of the five compared algorithm are shown in Table 2.

### 3.3. Performance Evaluation Criteria

With the purpose of conducting the experiments comprehensively, we introduce two measurements: detection power and running time. The detection power is defined below:

(6) $$\begin{array}{c} \text{Power}=\#\text{T}/\#\text{G}, \end{array}$$

where #G is the datasets that are generated by the same penetrance table (#G = 100 in the experiments) and #T is the number of datasets in which the two-locus SNP-SNP interaction associated with disease is detected.

### 3.4. Experiments on Simulated Datasets

**Figure 3:**
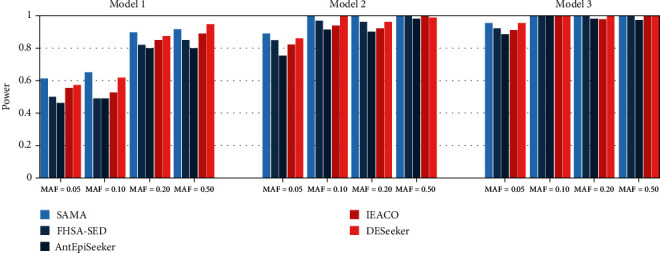
Power comparison of five compared algorithms on the datasets with 200 SNPs.

**Figure 4:**
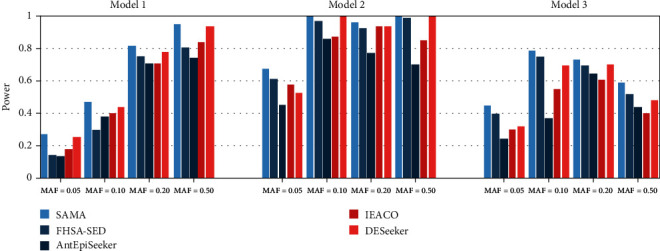
Power comparison of five compared algorithms on the datasets with 2000 SNPs.

Figures 3 and 4 present the detection power of the five compared algorithms on the three disease models. It is indicated from the figures that the SAMA algorithm is better than or equal to FHSA-SED, AntEpiSeeker, IEACO, and DESeeker on most settings, with the exception of MAF = 0.50 in Model 1 with 200 SNPs. SAMA detects all disease-associated SNP-SNP interactions on six settings for the datasets with 200 SNPs, and the algorithm detects all disease-associated SNP-SNP interactions on two settings for the datasets with 2000 SNPs. On the datasets with 200 SNPs, the other four algorithms can be comparable with SAMA because they also have good performance. On the datasets with 2000 SNPs, the detection power obtained by our algorithm is significantly greater than that of the other four algorithms, especially in Model 3. Followed by FHSA-SED and DESeeker, these two algorithms also show not bad performance. Next is IEACO. The performance of AntEpiSeeker performance is the worst in this experiment. The above analysis reveals that the proposed algorithm is more effective for detecting two-locus SNP-SNP interactions.

Tables 3 and 4 show the running time of the five compared algorithms on the three disease models. As illustrated in the two tables, the running time of our method is less than that of the other four methods. This demonstrates that SAMA can efficiently decrease the running time in detecting two-locus SNP-SNP interactions.

### 3.5. Experiments on a Real-World Biological Dataset

**Figure 5:**
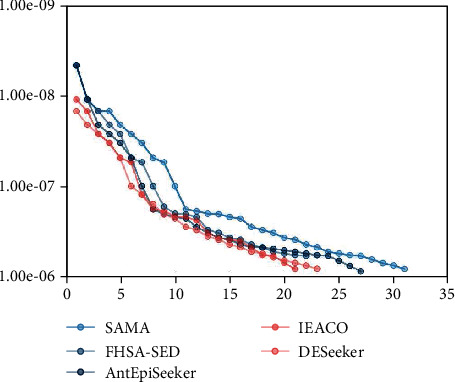
The number of two-locus SNP-SNP interactions detected by five algorithms.

According to the results of the simulated experiments, SAMA performs well for detecting two-locus SNP-SNP interactions. In this section, we conduct experiments on a real-world biological dataset [35]. The purpose of the experiment is to detect two-locus SNP-SNP interactions associated with the disease by using the five compared algorithms. The five algorithms are run 10 times, and Figure 5 is drawn according to the obtained p values. In the figure, a solid dot has two values, one is x-value, and the other is y-value. The y-value represents the p value, and the x-value denotes the SNP-SNP interaction detected by an algorithm with a certain p value. For the SAMA algorithm, 31 solid dots are detected, that is, 31 two-locus SNP-SNP interactions are detected. It can be seen evidently that the number of solid dots found by the proposed algorithm is more than that found by the other four algorithms. Followed by AntEpiSeeker, this algorithm detects 27 solid dots. Next is DESeeker and FHSA-SED. The DESeeker algorithm detects 23 solid dots, and the FHSA-SED algorithm detects 22 solid dots. The number of interactions found by IEACO is relatively less. This algorithm only finds 21 solid dots. The above analysis shows that SAMA can detect more two-locus SNP-SNP interactions than the other algorithms under the same number of iterations.

Table 5 presents the two-locus SNP-SNP interactions with p values less than 1.0e-06 detected by our method. In the table, the number of two-locus SNP-SNP interactions found by the SAMA algorithm with p values less than 1.0e-08, 1.0e-07, and 1.0e-06 are 1, 9, and 21, respectively.  Table 6 gives the number of two-locus SNP-SNP interactions detected by SAMA under different parameters. It can be seen from the Table 5 that rs380390 and rs1329428 are interacted with many other SNPs. The two SNPs are are located in the CFH gene, and the CFH gene has been commonly association with AMD [16, 38–40]. Furthermore, many SNPs included in detected SNP-SNP interactions are located in non-gene coding regions (NA). There are seven interactions between the CHF gene and NA when the p value is less than 1.0e-07, and there are ten interactions between the CHF gene and NA when the p value is between 1.0e-07 and 1.0e-06. The CHF gene has one interaction with the KDM4C gene, and it has two interactions with the MED27 gene. SNP rs2224762 is located in the KDM4C gene that can regulate chromosome segregation during mitosis [41]. This gene that may be associated with AMD has been reported before [22, 42]. SNPs rs7467596 and rs9328536 in the MED27 gene are related to melanoma [43], and the mutation in the MED27 gene may be associated with AMD [42]. Moreover, SAMA detected some new two-locus SNP-SNP interactions that have not been reported before. For example, rs1329428 has a interaction with rs10272438 and rs1740752 has a interaction with rs943008. SNP rs10272438 resides in the BBS9 gene which is involved in parathyroid hormone action in bones. SNP rs943008 resides in the NEDD9 gene, which is closely related to cancer. However, these two-locus SNP-SNP interactions require further examination in future studies. It can be seen from the Table 6 that the parameters we set before can find the most number of two-locus SNP-SNP interactions.

## 4. Conclusion

In the paper, we propose the SAMA algorithm to detect two-locus SNP-SNP interactions associated with disease. The global search ability of SAMA is greatly increased by using HC, DBM, and EC. The self-adaptive behavior of SLS enhances the local search ability of SAMA without significantly increasing the running time. When using simulated datasets, the experimental results indicate that SAMA is more effective than FHSA-SED, AntEpiSeeker, IEACO, and DESeeker in terms of detection power and running time. When utilizing the real-world biological dataset, the experiments show that the proposed algorithm successfully detected known disease-associated SNP-SNP interactions and some new suspected interactions. However, the SAMA algorithm still has some limitations. First, the detection power of SAMA is low for the disease models with small MAF. Furthermore, the current version of SAMA cannot detect high-order SNP-SNP interactions (SNPs > 2). As far as we know, there does not exist a powerful method for detecting high-order SNP-SNP interactions in GWAS. Therefore, detecting high-order SNP-SNP interactions associated with disease has many rooms to explore in the future.

## Figures and Tables

**Algorithm 1:**
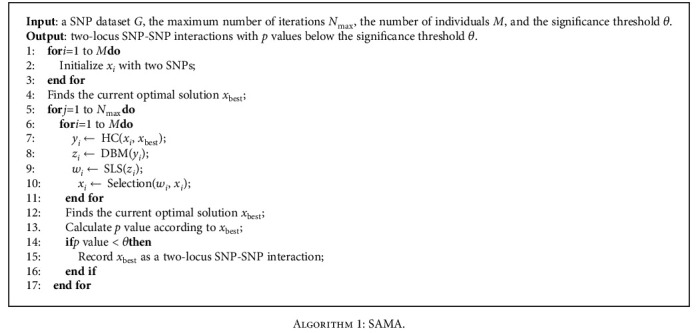
SAMA.

**Algorithm 2:**
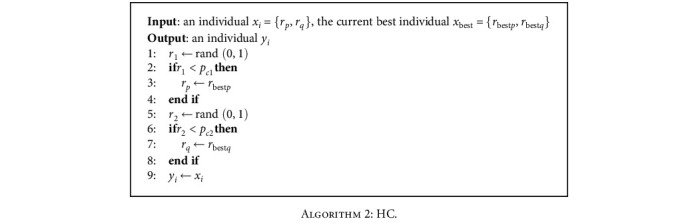
HC.

**Algorithm 3:**
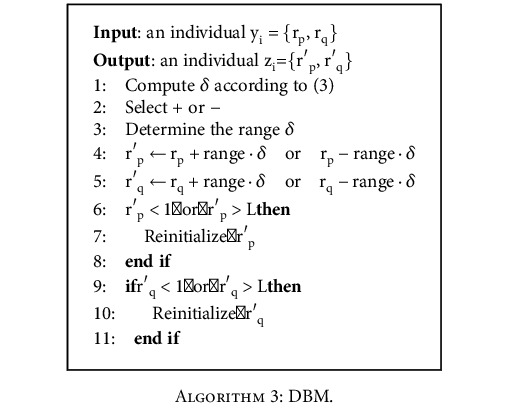
DBM.

**Algorithm 4:**
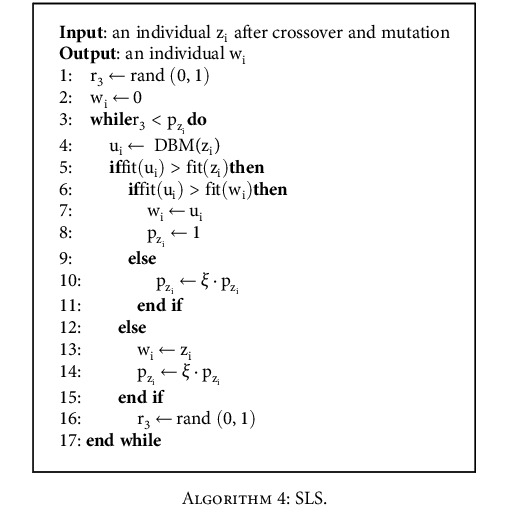
SLS.

**Table 1:** Details of three two-locus disease models.

MAF		0.05				0.10				0.20				0.50	
	AA	Aa	aa		AA	Aa	aa		AA	Aa	aa		AA	Aa	aa
Model 1 (P(D) = 0.1, h^2^ = 0.005)															
BB	0.098	0.098	0.098	BB	0.096	0.096	0.096	BB	0.092	0.092	0.092	BB	0.078	0.078	0.078
Bb	0.098	0.299	0.522	Bb	0.096	0.197	0.282	Bb	0.092	0.145	0.181	Bb	0.078	0.105	0.122
bb	0.098	0.522	0.912	Bb	0.096	0.282	0.408	Bb	0.092	0.181	0.227	Bb	0.078	0.122	0.142
Model 2 (P(D) = 0.1, h^2^ = 0.02)															
BB	0.096	0.096	0.096	BB	0.092	0.092	0.092	BB	0.084	0.084	0.084	BB	0.052	0.052	0.052
Bb	0.096	0.533	0.533	Bb	0.092	0.319	0.319	Bb	0.084	0.210	0.210	Bb	0.052	0.138	0.138
bb	0.096	0.533	0.533	Bb	0.092	0.319	0.319	Bb	0.084	0.210	0.210	Bb	0.052	0.138	0.138
Model 3 (P(D) = 0.1, h^2^ = 0.02)															
BB	0.080	0.192	0.192	BB	0.072	0.164	0.164	BB	0.061	0.146	0.146	BB	0.067	0.155	0.155
Bb	0.192	0.080	0.080	Bb	0.164	0.072	0.072	Bb	0.146	0.061	0.061	Bb	0.155	0.067	0.067
bb	0.192	0.080	0.080	Bb	0.164	0.072	0.072	Bb	0.146	0.061	0.061	Bb	0.155	0.067	0.067

**Table 2:** Parameter setting of five algorithms.

Algorithm	Parameters
SAMA	The crossover probabilities p_c1_ and p_c2_ = 0.8; the switch parameter *ξ* = 0.5; the number of individuals M = 500
FHSA-SED	The harmony memory considering rate HMCR =0.9; the pitch-adjusting rate PAR =0.35; the number of harmonies evaluated with Bayesian network scoring ‖HM1‖ = 250; the number of harmonies evaluated with Gini scoring ‖HM2‖ = 250
AntEpiSeeker	The size of large SNP sets largesetsize = 6; the size of small SNP sets smallsetsize = 3; the weight parameters *α* and *β* = 1; the pheromone evaporation rate *ρ* = 0.05; the initial pheromone *τ*_0_ = 100; the number of ants M = 500
IEACO	The switch parameter *θ* is 0.001; the upper bound of negative feedback pheromone on worse paths *μ* = 300; the weight parameters *α* and *β* = 1; the parameter determining the weight of negative feedback pheromone *γ* = 1; the number of ants M = 500
DESeeker	The number of SNPs in a large size SNP combination W = 6; the number of vectors M = 500

**Table 3:** Running time of five compared algorithms on the datasets with 200 SNPs.

Model	MAF	SAMA	FHSA-SED	AntEpiSeeker	IEACO	DESeeker
Model 1	0.05	9.12 ± 0.53	10.55 ± 0.59	46.63 ± 2.31	11.21 ± 0.76	10.03 ± 0.64
	0.10	8.97 ± 0.51	10.32 ± 0.53	48.52 ± 2.40	12.45 ± 0.81	9.89 ± 0.70
	0.20	9.32 ± 0.49	10.47 ± 0.58	47.71 ± 2.29	10.93 ± 0.79	9.93 ± 0.66
	0.50	9.55 ± 0.44	10.62 ± 0.62	45.63 ± 1.99	13.06 ± 0.82	10.32 ± 0.73

Model 2	0.05	9.53 ± 0.48	11.04 ± 0.65	48.57 ± 2.37	10.90 ± 0.71	10.54 ± 0.77
	0.10	9.29 ± 0.57	10.86 ± 0.68	49.12 ± 2.30	11.35 ± 0.66	9.98 ± 0.69
	0.20	8.86 ± 0.46	11.06 ± 0.64	46.83 ± 2.12	12.52 ± 0.73	10.74 ± 0.65
	0.50	9.22 ± 0.50	10.75 ± 0.70	46.89 ± 2.06	11.83 ± 0.68	9.76 ± 0.59

Model 3	0.05	9.06 ± 0.55	10.63 ± 0.63	50.02 ± 2.55	12.04 ± 0.74	10.63 ± 0.62
	0.10	9.52 ± 0.59	11.05 ± 0.68	47.74 ± 2.19	11.67 ± 0.80	10.72 ± 0.58
	0.20	9.32 ± 0.51	10.64 ± 0.57	48.82 ± 2.49	12.42 ± 0.69	9.48 ± 0.61
	0.50	9.94 ± 0.60	10.74 ± 0.61	45.90 ± 2.05	11.53 ± 0.78	9.80 ± 0.65

**Table 4:** Running time of five compared algorithms on the datasets with 2000 SNPs.

Model	MAF	SAMA	FHSA-SED	AntEpiSeeker	IEACO	DESeeker
Model 1	0.05	84.63 ± 3.76	98.74 ± 5.32	431.53 ± 11.57	108.64 ± 5.96	97.56 ± 4.97
	0.10	87.53 ± 4.02	103.63 ± 5.67	427.87 ± 10.94	109.42 ± 6.03	100.55 ± 5.17
	0.20	90.89 ± 3.90	98.85 ± 5.15	442.35 ± 10.52	111.34 ± 6.12	99.74 ± 5.20
	0.50	88.16 ± 3.95	101.15 ± 4.96	425.84 ± 12.02	104.44 ± 6.04	103.85 ± 5.06

Model 2	0.05	91.48 ± 4.12	97.88 ± 4.87	435.14 ± 12.53	110.45 ± 5.64	102.66 ± 5.07
	0.10	89.86 ± 3.79	100.56 ± 5.04	448.57 ± 10.89	102.63 ± 6.23	98.85 ± 5.12
	0.20	89.17 ± 4.03	99.95 ± 4.78	459.84 ± 11.78	101.34 ± 5.98	105.05 ± 5.31
	0.50	92.74 ± 3.87	100.13 ± 4.83	418.52 ± 10.97	105.65 ± 5.95	104.43 ± 5.13

Model 3	0.05	90.63 ± 3.93	97.73 ± 5.01	451.45 ± 12.32	112.56 ± 6.46	101.89 ± 5.44
	0.10	86.73 ± 3.89	103.54 ± 5.21	432.85 ± 11.67	109.93 ± 6.15	104.92 ± 5.19
	0.20	87.83 ± 4.07	96.97 ± 4.89	429.50 ± 12.02	113.56 ± 5.96	99.71 ± 5.08
	0.50	90.09 ± 3.86	101.34 ± 5.36	440.86 ± 12.63	114.37 ± 6.07	103.67 ± 5.32

**Table 5:** Results of two-locus SNP-SNP interactions detected by SAMA on AMD dataset.

SNP 1	Gene	SNP 2	Gene	p values
rs380390	CFH	rs1363688	NA	<1.0e-08
rs380390	CFH	rs2224762	KDM4C	
rs380390	CFH	rs555174	NA	
rs380390	CFH	rs1374431	NA	
rs380390	CFH	rs1740752	NA	

rs1329428	CFH	rs7467596	MED27	<1.0e-07
rs1329428	CFH	rs9328536	MED27	
rs1329428	CFH	rs3922799	NA	
rs1329428	CFH	rs10489076	NA	
rs1740752	N/A	rs3009336	NA	
rs380390	CFH	rs718263	NCALD	
rs380390	CFH	rs223607	NA	
rs380390	CFH	rs620511	NA	
rs380390	CFH	rs2178692	COPS7A	
rs380390	CFH	rs34512	NA	
rs380390	CFH	rs3853728	EGFEM1P	
rs380390	CFH	rs210758	NA	
rs380390	CFH	rs2446023	ZNF518A	
rs380390	CFH	rs2167167	NA	
rs380390	CFH	rs956275	PPAT	

rs380390	CFH	rs1896373	NA	<1.0e-06
rs380390	CFH	rs1896373	NA	
rs380390	CFH	rs143627607	DDX3X	
rs1329428	CFH	rs10504043	ANK1	
rs1329428	CFH	rs10272438	BBS9	
rs1329428	CFH	rs2695214	PPP3CA	
rs1329428	CFH	rs78812154	NA	
rs1329428	CFH	rs74412587	NA	
rs1329428	CFH	rs1363688	NA	
rs1329428	CFH	rs9328536	MED27	
rs1740752	NA	rs943008	NEDD9	

**Table 6:** Number of two-locus SNP-SNP interactions detected by SAMA under different parameters.

*ξ*	0.1	0.2	0.3	0.4	0.5	0.6	0.7	0.8	0.9
p_c1_ and p_c2_									
.1	9	12	14	17	19	18	17	13	10
.2	12	14	17	20	23	21	18	16	11
.3	13	13	16	19	21	18	20	16	13
.4	13	15	16	20	24	21	21	18	18
.5	16	17	17	23	30	25	23	20	19
.6	15	17	18	24	28	25	25	22	17
.7	15	13	18	25	27	26	27	21	19
.8	14	14	22	28	31	30	27	25	26
.9	12	13	17	23	29	25	26	22	21

## Data Availability

The data used to support the findings of this study are included within the article, which are described in detail in [30, 32], respectively.

## Abbreviations

ACO: Ant colony optimization
AntEpiSeeker: Two-stage ant colony optimization algorithm
AMD: Age-related macular degeneration
DE: Differential evolution
DBM: Distributed breeder mutation
DESeeker: Two-stage differential evolution algorithm
ES: Elitist selection
FHSA-SED: Harmony search algorithm with two scoring functions
GA: Genetic algorithm
GWAS: Genome-wide association study
IEACO: Self-adjusting ant colony optimization based on information entropy
HC: Hybrid crossover
LS: Local search
MA: Memetic algorithm
MAF: Minor allele frequency
SAMA: Self-adaptive memetic algorithm
SNP: Single-nucleotide polymorphism
SLS: Self-adaptive local search.
